# Characterization of the complete mitochondrial genome of *Triceratopyga calliphoroides* (Rohdendorf, 1931) (Insecta: Diptera: Calliphoridae)

**DOI:** 10.1080/23802359.2019.1624218

**Published:** 2019-07-10

**Authors:** Mustafa Zafer Karagozlu, Seong Hwan Park, Sang-Eon Shin, Chang-Bae Kim

**Affiliations:** a Department of Biotechnology, Sangmyung University, Seoul, Korea;; b Department of Legal Medicine, Korea University College of Medicine, Seoul, Korea

**Keywords:** Insecta, Diptera, Calliphoridae, complete mitochondrial genome, *Triceratopyga calliphoroides*

## Abstract

*Triceratopyga calliphoroides* is a blowfly species which is the only member of the genus *Triceratopyga*. Because of their forensic importance, we sequenced the complete mitogenome of the *T. calliphoroides* and analyzed phylogenetic relationships. According to data, it has the longest mitogenome in the family with 16,529 bp in length. In the phylogenetic tree, *T. calliphoroides* were positioned in the subfamily Calliphorinae, and the closest species is *Caliphora vomitoria*. This is the first complete mitogenome record for the species.

*Triceratopyga calliphoroides* is a common and synanthropic blowfly species in eastern Asia. It is the only member of the genus *Triceratopyga.* Since they occur in human cadavers, they have forensic importance for the determination of the post-mortem interval (Zhang et al. 2014). In this study, we sequenced and analyzed the first complete mitogenome of *T. calliphoroides*. After sequencing of the miotogenome, phylogenetic relationships of the genus *Triceratopyga* in the family Calliphoridae were investigated.

The *T. calliphoroides* specimen was collected from the forest, Mt. Bukhan, Seoul/37°65′03.6″N 127°00′89.1″E, May 2016. The specimens deposited in the Department of Legal Medicine, Korea University (16Cl11). After species identification, the total DNA extracted from the legs and thorax, paired-end reads of mitogenome were generated by Illumina Miseq sequencing system. The reads were targeted for reconstructing of the complete mitogenome by MITObim (Hahn et al. 2013). The circular sequence was annotated using the MITOS web server (Bernt et al. 2013) and Geneious 11 software (Kearse et al. 2012).

The length of complete the mitochondrial genome is 16,529 bp in length which is the longest mitogenome recorded in the family (GeneBank accession number MK893471). Typically it contains 13 protein-coding genes, 22 transfer RNAs, two ribosomal RNAs, and an AT rich region. There are 13 overlapping regions in the genome show length variation ranging from 1 to 8 bp while there are 15 intergenic sequences show length variation ranging from 1 to 18 bp. The longest intergenic sequence is between tRNA-Glu and tRNA-Phe genes, and the longest overlapping region is between tRNA-Trp and tRNA-Cys.

According to the nucleotide sequence of concatenated 13 protein-coding genes, the phylogenetic tree of the family Calliphoridae reconstructed (Figure 1). For reconstruction, 24 mitogenome records from the family Calliphoridae and two records from the family Sarchopagidae were retrieved from the GenBank. Although there were more mitogenome records from the family Calliphoridae, most records belonged to the same species. For the analysis, we randomly chose the one individual record from the multiple recorded species. In the phylogenetic tree, *T. calliphoroides* were positioned in the subfamily Calliphorinae, and the closest species is *Caliphora vomitoria.* Besides, all subfamilies of the family Calliphoridae were monophyletic. The previous study nuclear (28S rRNA and ITS2) and mitochondrial (COI and 16S rRNA) markers combination based study also suggested similar results (Marinho et al. 2012). However, another study which was based on one mitochondrial (COI) and three nuclear (CPS, EF1a, and 28S rRNA) suggested that the subfamily Melanomyinae nested in the subfamily Calliphorinae. Hence Calliphorinae is not monophyletic (Singh and Wells 2013). Since there is no complete mitochondrial genome recorded from the subfamily Melanomyinae, we cannot confirm the relationship of these two subfamilies. Besides, Calliphorinae and Luciliinae were clustered together, and the clade consist of these two subfamilies has a sister group relationship with the subfamily Chrysomyinae. These findings are similar to previous studies (Marinho et al. 2012; Singh and Wells 2013). This study provides additional data for the investigation of the Calliphoridae phylogeny.

**Figure 1.**
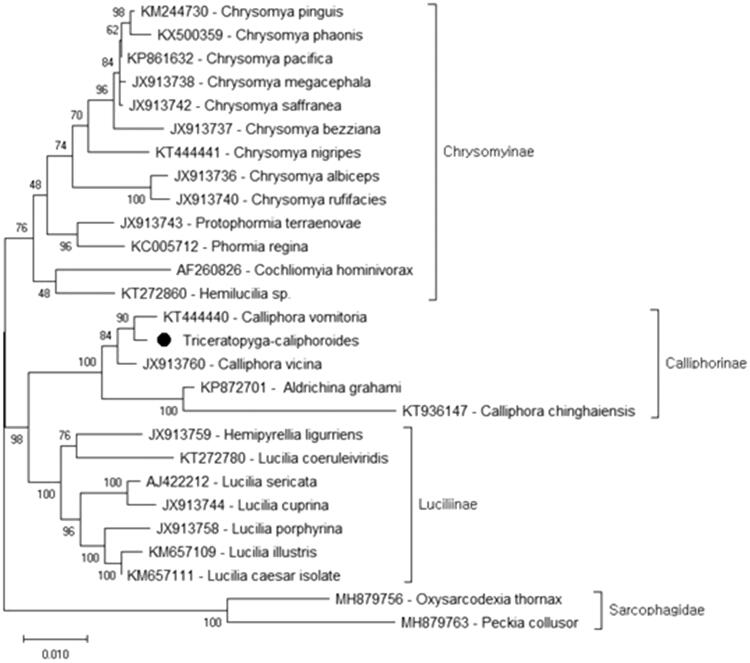
The molecular phylogeny of *Triceratopyga calliphoroides* (black circle) in the family Calliphoridae was analyzed with a maximum likelihood statistical method using MEGA X (Kumar et al. 2018). mtREV with Freqs (+F) model used for amino acid substitution and bootstrap method replicated 1000 times for the test of the phylogeny. For reconstruction, the complete mitochondrial genomes of the species were retrieved from the GenBank and nucleotide sequences of all protein-coding genes were used for analysis. The species *Peckia collusor* and *Oxysarcodexia thornax* from the family Sarcophagidae were used as outgroup.
